# A Chitosan-Based Flocculation Method for Efficient Recovery of High-Purity B-Phycoerythrin from a Low Concentration of Phycobilin in Wastewater

**DOI:** 10.3390/molecules28083600

**Published:** 2023-04-20

**Authors:** Yingye Liang, Luming Deng, Zhenhui Feng, Qianqian Ouyang, Xia Wu, Weiyan Quan, Yuzhen Zhu, Hua Ye, Kefeng Wu, Hui Luo

**Affiliations:** 1 Marine Biomedical Research Institution, Guangdong Medical University, Zhanjiang 524023, China; isliangyy@163.com (Y.L.); 13760779214@163.com (L.D.); fengzhlcy@163.com (Z.F.); oyqq617@gdmu.edu.cn (Q.O.); wx19825558302@163.com (X.W.); luohui@gdmu.edu.cn (H.L.); 2 Guangdong (Zhanjiang) Provincial Laboratory of Southern Marine Science and Engineering, Zhanjiang 524023, China; zhuyuzhen2003@126.com; 3 Zhanjiang Engineering Research Center for Algae High-Value Utilization, Zhanjiang 524023, China; 4 The Marine Biomedical Research Institute of Guangdong Zhanjiang, Zhanjiang 524023, China

**Keywords:** chitosan, flocculation, B-phycoerythrin, phycobilin in wastewater, economic evaluation

## Abstract

Increasing the yield and purity of B-phycoerythrin (B-PE) can improve the economic state of microalgae industrial processing. One method of cost reduction involves the recovery of remaining B-PE from wastewater. In this study, we developed a chitosan (CS)-based flocculation technique for the efficient recovery of B-PE from a low concentration of phycobilin in wastewater. We investigated the effects of the molecular weight of chitosan, B-PE/CS mass ratio, and solution pH on the flocculation efficiency of CS and the effects of phosphate buffer concentration and pH on the recovery rate of B-PE. The maximum flocculation efficiency of CS, recovery rate, and purity index of B-PE were 97.19% ± 0.59%, 72.07% ± 1.37%, and 3.20 ± 0.025 (drug grade), respectively. The structural stability and activity of B-PE were maintained during the recovery process. Economic evaluation revealed that our CS-based flocculation method is more economical than the ammonium sulfate precipitation method is. Furthermore, the bridging effect and electrostatic interaction play important roles in B-PE/CS complex flocculation process. Hence, our study provides an efficient and economical method to recover high-purity B-PE from a low concentration of phycobilin in wastewater, which promoted the application of B-PE as a natural pigment protein in food and chemical applications.

## 1. Introduction

B-phycoerythrin (B-PE) is the main phycobiliprotein of the *Porphyridium* species, consisting of *α*, *β*, and *γ* subunits, with a total molecular weight of 263 kDa [1,2,3,4]. B-PE usually has two absorption peaks at ~545 and ~565 nm, and a shoulder peak at ~498 nm, and a fluorescence emission maximum at 580 nm [5]. Recently, B-PE has received massive amounts of attention from the scientific community owing to its extensive applications in food, pharmaceuticals, cosmetics, textiles, and as printing dyes [6,7]. Because of the high economic value of B-PE, large-scale preparation from *Porphyridium* species is required [5,8]. Currently, industrial extraction strategies for B-PE are primarily focused on the enrichment of phycobilisomes, and then obtaining B-PE from high concentrations of phycobilisome solution [9,10]. However, during the harvesting process, mechanical forces, such as centrifugation and filtration, can destroy the integrity of phycobilisomes and release phycobiliproteins into the media, resulting in the waste of phycobiliprotein resources [11]. B-PE is, in fact, present in waste media at very low concentrations. However, to the best of our knowledge, no efficient methods have been reported heretofore for the recovery of B-PE from a low concentration of phycobilin in wastewater.

Recently, many methods have been proposed for phycobiliprotein extraction from the phycobilisome solution, including ammonium sulfate precipitation [12], ultrafiltration [13], chromatography [5], and aqueous two-phase systems [14], among others. However, these methods are applicable only to a high concentration of the phycobilisome solution. Ammonium sulfate precipitation is the most frequently employed method for precipitating phycobiliproteins. However, the complexity and the lack of selectivity of operating procedures limits its application [12,15,16]. Ultrafiltration is another commonly used method for the enrichment of phycobiliproteins, but it requires a lot of time and energy [17]. From an economic perspective, chemical precipitation and ultrafiltration are not efficient and effective with regard to energy or cost when the concentration of phycobiliproteins is less than 0.1 g/L. Thus, it remains a substantial challenge to obtain the desired B-PE product from a low concentration of phycobilin in wastewater.

Chitosan (CS) is a polysaccharide consisting of *β*-linked D-glucosamine and *N*-acetyl-D-glucosamine [18,19]. CS is derived from the deacetylation of chitin, a polymer obtained from the exoskeleton of shrimp, crab, or other crustaceans [20]. In addition, the cell wall of the filamentous fungi is also a good source of CS [21]. Notably, CS can be dissolved in aqueous acidic media (glacial acetic acid, hydrochloric acid, etc.) through the protonation of primary amines. Generally, the solubility of CS depends on its degree of deacetylation (DD) and molecular weight (MW), with a pKa value of around 6.5 [22,23].

CS has attracted enormous amount of attention from the biomedical community worldwide due to its intrinsic safety, recyclability, and biocompatibility [18,24,25]. The authors of previous studies reported that CS can not only adsorb and remove harmful substances from water, but it can also kill bacteria [26]. Therefore, it is an ideal adsorbent for the purification of drinking water. As well as adsorbing suspended particles, dyes, and heavy metals, CS reduces environmental contamination and waste disposal costs [27,28]. Further, CS can be easily processed into different forms for biomedical applications such as tissue engineering and wound healing [29,30]. In bone tissue engineering, CS often requires to be combined with hydroxyapatite to sufficiently improve its engineering behavior such as biocompatibility and integration of hard tissue [31,32]. In skin tissue engineering, suitable chitosan-based biomaterials were developed through preparation techniques such as freeze drying, electrospinning, and so on [33].

Moreover, the use of biopolymer-based flocculants has been proposed as a feasible approach to remove proteins from different solutions. The authors of previous studies found that cationic CS interacted electrostatically with proteins to form CS–protein complexes, which is beneficial to achieve efficient solid–liquid separation [34,35]. However, most of the above studies addressed the problem of deproteinization in high-protein-concentration solutions, but they were rarely concerned with the recovery of proteins, especially for B-PE [36,37]. Further investigation demonstrated that a wide range of flocculation factors, such as the MW of macromolecules, biopolymer ratio, concentrations, pH, and ionic strength, influenced the stability of CS–protein complexes [34,38]. Thus, research on the properties of B-PE/CS complexes could provide valuable insights into the recovery of B-PE from wastewater.

To address the challenge of improving the stability of phycoerythrin, oligochitosan has been used as a flocculant to promote the formation of phycoerythrin–oligochitosan complexes from high concentrations of phycoerythrin in a solution [3]. However, its potential for recovering B-PE from a low concentration of phycobilin in wastewater has not been reported so far. Additionally, the convenience of the recovery of B-PE is also an important consideration. Therefore, the aim of the present study was to develop an efficient and economical method for recovering B-PE from a low concentration of phycobilin in wastewater using CS as a flocculant. We studied the effects of different flocculation factors, namely, the MW of CS, B-PE/CS mass ratio, and solution pH on the flocculation efficiency of CS. We also investigated the effects of phosphate buffer concentration and pH on the recovery rate of B-PE to determine the optimized recovery conditions. We further performed an economic evaluation of our CS-based flocculation method using the ammonium sulfate precipitation method as a positive control. Meanwhile, we characterized the properties of the recovered B-PE. Herein, we discuss the flocculation mechanism of B-PE/CS complexes, which could provide the basis for the formation of B-PE/CS complexes.

## 2. Results and Discussion

### 2.1. Formation of B-PE/CS Complexes

B-PE with an MW of approximately 280 kDa maintains stability in the pH range of 4.0–10.0 [2,39]. When the pH value is greater than its isoelectric point (pI, 4.39), B-PE is negatively charged, and it can interact electrostatically with positively charged CS to form B-PE/CS complexes [40]. In light of the above findings, an ideal strategy for the recovery of B-PE from a low concentration of phycobilin in wastewater using cationic flocculants such as CS was proposed. In addition, all experiments were carried out at room temperature to reduce the costs.

#### 2.1.1. Effect of MW of CS

**Figure 1:**
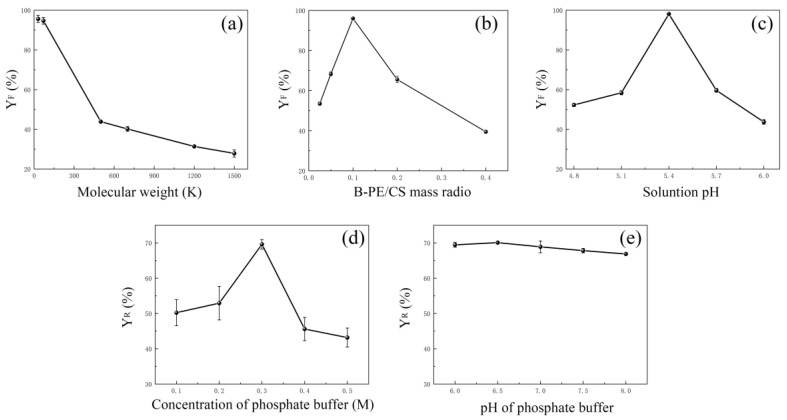
The effects of different factors on flocculation efficiency: (**a**) MW of CS; (**b**) B-PE/CS mass ratio; (**c**) solution pH. The effects of different factors on recovery rate: (**d**) concentration of phosphate buffer; (**e**) pH of phosphate buffer.

The authors of previous studies discovered that the flocculation effect of CS was directly associated with its MW [34,41]. To investigate the effect of MW of CS on flocculation efficiency, six CS samples with MW values ranging from 30 to 1500 kDa were selected as flocculants. The results show that the flocculation efficiency of CS decreased significantly from 95.61% ± 1.82% to 27.83% ± 1.78% (*p* < 0.05) as the MW gradually increased from 30 to 1500 kDa (Figure 1a). Therefore, 30 kDa is regarded as the optimal MW. As shown in Figure 1a, when the MW of CS exceeded 500 kDa, an inflection point appeared. At the inflection point, the flocculation efficiency of CS with an MW of 500 kDa was only 43.87% ± 0.32%, which was less than half of that of CS with an MW of 30 kDa. The above results suggested that very high-molecular-weight CS (over 500 kDa) was difficult to form stable complexes with B-PE. However, this trend was inconsistent with some previous studies, the authors of which reported that increasing the MW of CS would improve the flocculation efficiency of CS on oyster total hydrolysates and bentonite suspensions [35,41]. Although the MW of CS played a key role in B-PE/CS flocculation, the underlying mechanism was still unclear. A study found that high-molecular-weight CS easily became entangled because of the formation of intermolecular hydrogen bonds between the hydroxyl and amino groups, resulting in there being fewer active sites available for the adsorption of B-PE [34]. Therefore, CS with appropriate MW should be selected for each specific raw material.

#### 2.1.2. Effect of B-PE/CS Mass Ratio

Besides the effect of the MW of CS on flocculation efficiency, the effect of B-PE/CS mass ratio was investigated. Based on the above results, CS with an MW of 30 kDa was used as a flocculant. As depicted in Figure 1b, when the B-PE/CS mass ratio was 0.1, B-PE was almost completely adsorbed by CS. Therefore, the flocculation efficiency of CS reached its maximum, which could be attributed to the fact that when the B-PE/CS mass ratio was less than 0.1, an overdose of CS resulted in a large number of positive charges, and it was then adsorbed on the surface of the charge-neutralized particles. As a result, the particles carried positive charges. Subsequently, the positively charged particles repulsed each other and caused re-stabilization [42,43]. However, as the B-PE/CS mass ratio further increased above 0.1, the flocculation efficiency of CS decreased significantly. This may be because there were not enough charged reactive sites for CS to interact electrostatically with B-PE, and the bridging effect of CS decreased with the increase in the B-PE/CS mass ratio [38]. On the whole, the B-PE/CS mass ratio of 0.1 was determined as the optimal ratio for further testing.

#### 2.1.3. Effect of Solution pH

As the electrostatic interaction between B-PE and CS was closely related to the solution pH, we deemed it necessary to carefully investigate the effect of the solution pH on the flocculation efficiency. The authors of studies have evidenced that the pI of protein was associated with CS flocculation, which was achieved by adjusting the strength of the electrostatic interaction of ionizable groups [38,40]. As B-PE carried negative charges only at pH values greater than its pI (4.39) and CS dissolved only in acidic solutions, flocculation between B-PE and CS occurred at a narrow pH range (4.8–6.0). As evident in Figure 1c, flocculation between the two biopolymers was observed in the pH range of 4.8–6.0, and the maximum flocculation efficiency of CS was obtained at a pH of 5.4 (*p* < 0.05). Notably, this pH range was similar to that of the soybean protein isolate (SPI)–CS coacervation, in which the pI of SPI (4.8) was very close to that of B-PE [40]. However, for a pH that is greater or lower than 5.4, the flocculation efficiency of CS decreased significantly owing to the weak electrostatic interaction between B-PE and CS. Thus, a pH value of 5.4 was deemed to be optimal.

### 2.2. Dissociation of B-PE/CS Complexes

The release of B-PE from B-PE/CS complexes was a key step in the recovery of B-PE. To efficiently release B-PE from B-PE/CS complexes, phosphate buffer was used to recover B-PE. According to the extant literature, salt concentration and pH may have an effect on the stability of B-PE/CS complexes [38,44]. Thus, it is critical to select an appropriate phosphate buffer as a dissociation solution to release B-PE from B-PE/CS complexes.

The recovery rate of B-PE increased upon increasing the concentration of phosphate buffer from 0.1 M for 50.22% ± 3.71% to 0.3 M for 69.60% ± 1.34% (*p* < 0.05; Figure 1d). However, the recovery rate of B-PE significantly decreased as the concentration of phosphate buffer increased above 0.3 M, which could be ascribed to the increased difficulty in releasing B-PE from B-PE/CS complexes because of the salting-in effect [45]. Furthermore, we found no significant difference in the recovery rate of B-PE when the pH of phosphate buffer was 6.0–8.0, with a maximum rate reached at pH 6.5 (Figure 1e). Finally, the phosphate buffer concentration and pH of 0.3 M and 6.5, respectively, were determined to be the optimal dissociation conditions. These findings suggested that the recovery rate of B-PE released from B-PE/CS complexes was mainly related to the ionic strength in neutral phosphate buffer, but not to the pH of phosphate buffer.

### 2.3. Analysis of B-PE Precipitation

**Figure 2:**
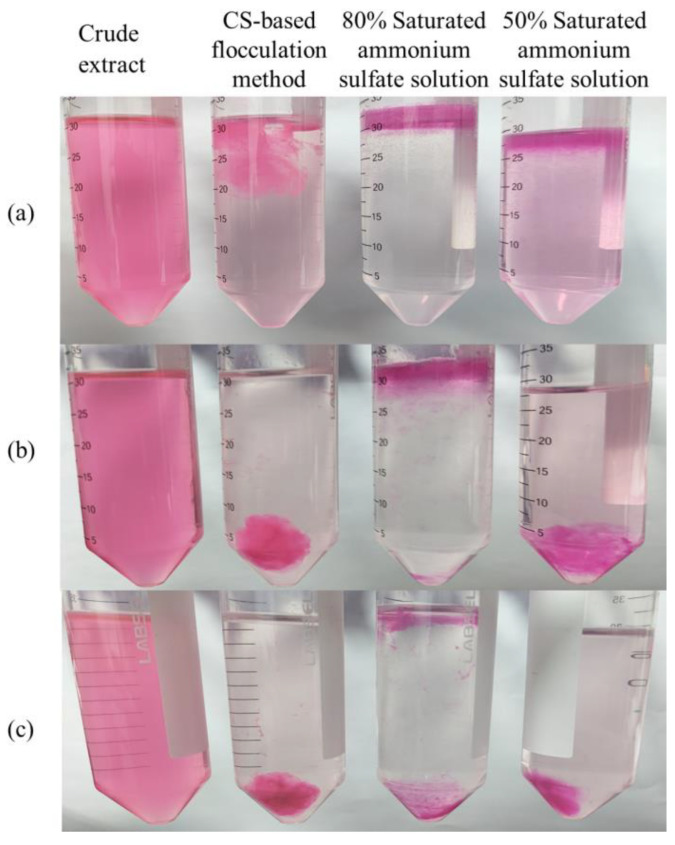
Comparison between CS-based flocculation method and ammonium sulfate precipitation method. (**a**) Image before centrifugation; (**b**) image after centrifugation at 2000 rpm for 15 min; (**c**) image after centrifugation at 8000 rpm for 30 min.

Based on the requirements of convenience and economy of industrial applications, precipitation methods have attracted a lot of attention as the most cost-effective methods of protein engineering [15,45]. Therefore, the ammonium sulfate precipitation method was used as a positive control to evaluate the flocculation efficiency of the CS-based flocculation method. As shown in Figure 2a, all precipitants could form complexes with B-PE. To further evaluate the purity index and the recovery rate of B-PE, centrifugation was used as an important tool to precipitate the complexes formed by our CS-based flocculation method and the ammonium sulfate precipitation method.

As depicted in Figure 2b,c, B-PE/CS complexes could be completely precipitated from wastewater by centrifugation at 2000 rpm for 15 min or 8000 rpm for 30 min. As shown in Table 1, under the condition of centrifugation at 2000 rpm for 15 min, the purity index and recovery rate of B-PE obtained by the CS-based flocculation method were 3.20 ± 0.025 (drug grade) and 72.07% ± 1.37%, respectively. When ammonium sulfate saturation was 80%, B-PE with a fine granular form was enriched onto the solution surface because of the salting-out effect [45]. However, the granular B-PE precipitation could be hardly precipitated by centrifugation at 2000 rpm for 15 min (Figure 2b). As shown in Figure 2b,c, in the 50% saturated ammonium sulfate solution, B-PE solubility increased at low ionic strength due to the salting-in effect. Thus, it was difficult to precipitate from saturated ammonium sulfate solution even by centrifugation [45]. Therefore, the recovery rate of B-PE in the 50% saturated ammonium sulfate solution was 36.13% ± 2.44%, which was markedly lower than 45.10% ± 2.28% in the 80% saturated ammonium sulfate solution (Table 1). By comparison, the recovery rate of B-PE in the optimal CS solution (CS with an MW of 30 kDa, B-PE/CS mass ratio of 0.1, and pH of 5.4) was much higher than that of B-PE in the 50% and 80% saturated ammonium sulfate solutions. Additionally, the purity index values of B-PE precipitated from 80% and 50% saturated ammonium sulfate solutions were approximately one-fifth and one-fourth that of B-PE recovered by the optimal CS solution (Table 1). Moreover, using salting out to purify proteins also caused contaminants to frequently precipitate with the protein of interest [45]. Hence, the above results suggest that our CS-based flocculation method could recover high-purity B-PE from a low concentration of phycobilin in wastewater more efficiently than the ammonium sulfate precipitation method can.

### 2.4. Economic Evaluation

Economic evaluation plays a pivotal role in controlling the total costs and improving the economic benefits of any method [8,46]. In this study, considering the purity index and recovery rate of B-PE obtained by the ammonium sulfate precipitation method, the 80% saturated ammonium sulfate solution was selected as a positive control to evaluate the economics of our CS-based flocculation method.

**Figure 3:**
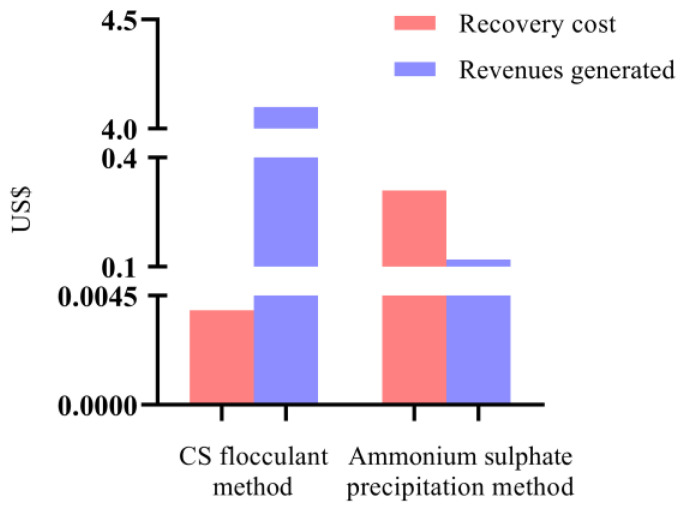
Economic evaluation for CS-based flocculation method and ammonium sulfate precipitation method.

Under optimal recovery conditions, the total costs of our CS-based flocculation method were markedly lower than that of the ammonium sulfate precipitation method (nearly 1/80th; Figure 3 and Table 2). In terms of material costs, the current price of CS (approximately USD 280 per kg) was much higher than the price of ammonium sulfate (approximately USD 10 per kg). Although the unit price of CS was much higher than that of ammonium sulfate, the amount of CS used (1.39 × 10^−5^ kg) in the recovery process was approximately 2230.22 times less than that of ammonium sulfate (3.10 × 10^−2^ kg), which is conducive to reducing the total costs. The above results imply that the unit price of CS is not the only factor determining the total costs. The costs are also related to the amount of CS used.

In this study, the purity index of B-PE was found to be positively correlated with the revenues generated (Table 2). By using 100% of material costs as the baseline, the revenues generated by B-PE were more than 34.17 times higher than that of B-PE obtained by the ammonium sulfate precipitation method because of the higher purity of B-PE recovered by the CS-based flocculation method under optimal recovery conditions.

### 2.5. Properties of the Recovered B-PE

**Figure 4:**
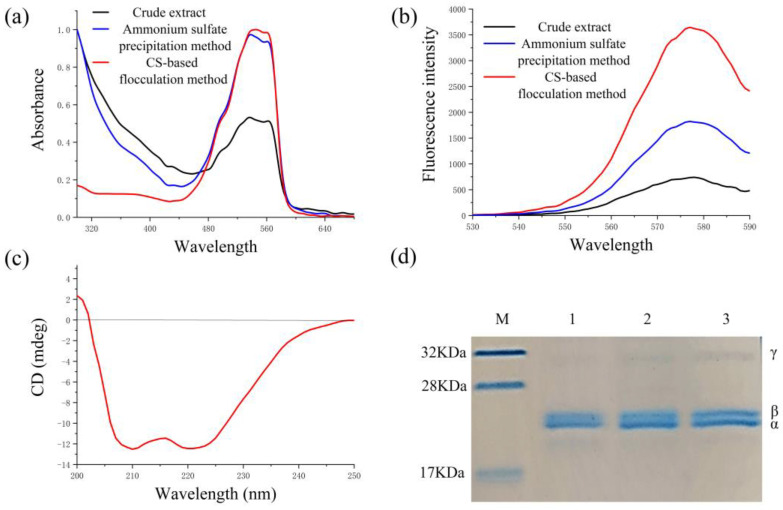
Properties of the recovered B-PE. (**a**) UV spectra of crude extract, B-PE sample recovered by ammonium sulfate precipitation method, and CS-based flocculation method; (**b**) fluorescence emission spectra of crude extract, B-PE sample recovered by ammonium sulfate precipitation method, and CS-based flocculation method; (**c**) CD spectrum of B-PE sample recovered by CS-based flocculation method; (**d**) SDS-PAGE, 1: crude extract; 2: B-PE sample recovered by ammonium sulfate precipitation method; 3: B-PE sample recovered by CS-based flocculation method.

The structural stability and activity of B-PE were the key parameters used to evaluate the feasibility of the recovery process of B-PE. The recovered B-PE had two absorption peaks at 545 and 565 nm and a shoulder peak at 498 nm (Figure 4a), which was consistent with the typical absorption spectrum of B-PE that was reported previously [5]. In addition, the fluorescence emission spectra of the recovered B-PE displayed a maximum fluorescence level at 578 nm when it was excited at 498 nm (Figure 4b). These results indicate that the recovered B-PE retained its native conformation and fluorescence properties during the recovery process, which is in agreement with the results of similar studies reported [2,5].

The circular dichroism (CD) spectrum of the recovered B-PE exhibited two negative minima in the ultraviolet region at 208 and 222 nm (Figure 4c), suggesting that the secondary structure of the recovered B-PE was α-helix. The obtained results are consistent with previous studies that analyzed the properties of B-PE [14,47,48].

In SDS-PAGE, three major separated bands were found in the lanes (Figure 4d). Two bulky bands between 17 and 28 kDa were assigned to the α and β subunits of B-PE, while another band at 32 kDa was attributed to the γ subunit of B-PE. These values were consistent with those that have reported been previously [3,5]. Altogether, the above results suggested that the structural stability and activity of B-PE were maintained during the recovery process.

### 2.6. Analysis of B-PE Binding with CS

**Figure 5:**
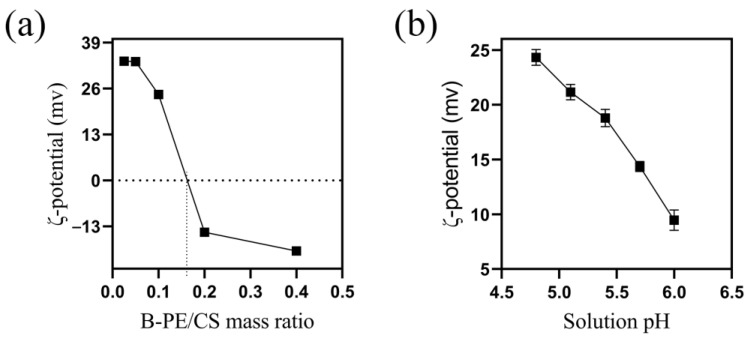
The factors effected ζ potential: (**a**) B-PE/CS mass ratio; (**b**) solution pH.

ζ potential changes were analyzed to anticipate the suspension’s stability, which contributed to clarifying the flocculation mechanism [49]. As shown in Figure 5a, the ζ potential of the B-PE solution remained almost unchanged (about 33 mV) when the B-PE/CS mass ratio was below 0.05, which indicates that the ζ potential reached a plateau [50]. This was associated that the overdosed CS-enhanced re-stabilization of the suspensions, decreasing the flocculation efficiency and hindering floc formation. The ζ-potential of suspensions changed from 24.3 ± 0.99 to −14.67 ± 0.33 as the B-PE/CS mass ratio increased from 0.1 to 0.2. Due to the increased amount of chitosan covering the B-PE surface, the collision rate was increased by the reduced electrostatic potential at the surface of the B-PE, resulting in flocculation and precipitation. Chitosan-free patches on the surface allowed the chitosan molecules to attach simultaneously to more than one B-PE micelle upon contact to cause bridging flocculation, which induced the phase separation observed at low chitosan concentrations [51,52].

In addition, the ζ potentials changed from 14.33 ± 0.59 to 9.48 ± 0.77 as the solution pH increased from 4.8 to 6.0 (Figure 5b). The ζ potentials of suspensions were positive, regardless of the pH value. The results indicated that the process of B-PE/CS flocculation began before the isoelectric point was reached. Thus, flocculation with CS occurred in a fairly broad range of variations in the particle electrokinetic potential instead of in the isoelectric state alone, as was described for the charge neutralization mechanism of flocculation [51,52]. The results suggested that charge neutralization was also contributed to the flocculation process.

**Figure 6:**
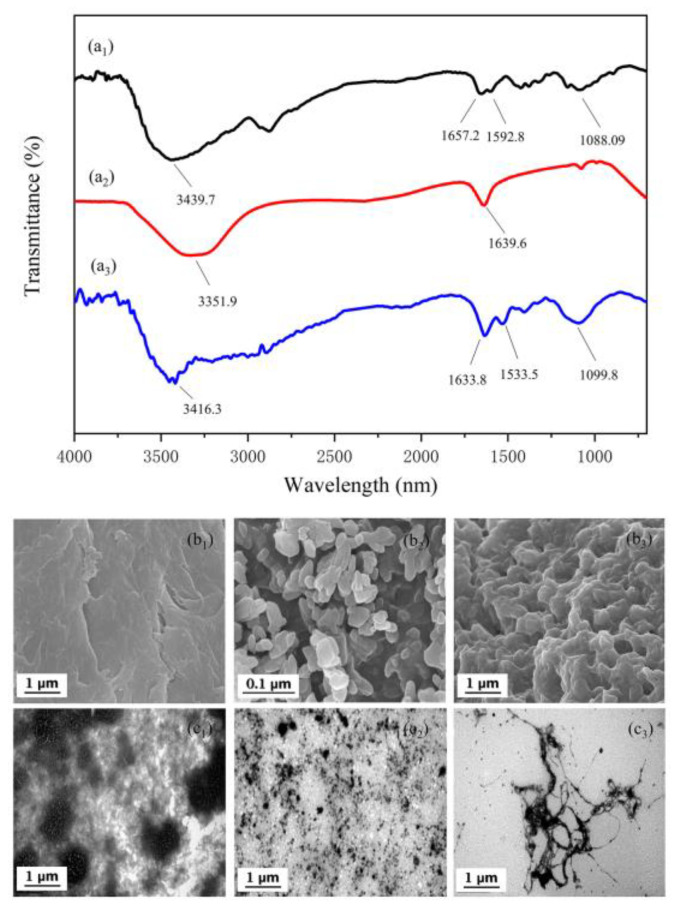
FTIR: (**a_1_**) CS; (**a_2_**) B-PE; (**a_3_**) B-PE/CS complexes. SEM: (**b_1_**) CS; (**b_2_**) B-PE; (**b_3_**) B-PE/CS complexes. TEM: (**c_1_**) CS; (**c_2_**) B-PE; (**c_3_**) B-PE/CS complexes.

The changes in wavelength and stretching vibration intensity of absorption bands in the FTIR spectrum were used to analyze the interaction between B-PE and CS. As depicted in Figure 6(a_1_–a_3_), the FTIR spectrum of CS showed a peak at 3439.7 cm^−1^, belonging to the stretching vibration of −NH groups and −OH groups [53]. The peaks at 1592.8 and 1088.1 cm^−1^ correspond to the N−H bending vibrations and C−O stretching vibrations, respectively [53,54]. In addition, the absorption band at 1657.2 cm^−1^ indicates the presence of the −CONH_2_ groups [40]. For the FTIR spectrum of B-PE, the typical characteristic peaks of B-PE were at 3351.9 and 1639.6 cm^−1^, belonging to O−H stretching vibration and C = O stretching vibration (free carboxyl groups), respectively [3,40]. By comparing the spectra of B-PE/CS complexes and B-PE and CS, the broad bands of CS and B-PE at 3439.7 cm^−1^ and 3351.9 cm^−1^, respectively, were founded to be shifted to 3416.3 cm^−1^, implying the occurrence of electrostatic interaction between B-PE and CS. Specifically, functional groups, including quaternary ammonium structure in CS and carboxyl in B-PE, contributed to the flocculation process. Furthermore, the peak shape of B-PE/CS complexes at 1099.8 cm^−1^ became broader, and the absorption strength increased, indicating that the hydrogen bonding of B-PE/CS complexes was enhanced compared with those of B-PE and CS [3].

The SEM results are illustrated in Figure 6(b_1_–b_3_). We found a huge difference between the surface morphology of CS, B-PE, and B-PE/CS complexes. CS had a plicate sheet-like structure and rough surface, providing a larger specific surface area for flocculating proteins [55]. In addition, numerous spherical particles and amorphous materials were observed in B-PE by SEM. Notably, the B-PE/CS complexes exhibited a cross-linked and tight, reticulated structure, which was due to the bridging effect of CS to form larger particles with an obvious long chain, indicating that the bridging effect played a major role in the B-PE/CS complex flocculation process. This finding is congruent to those observed in previous studies [38,56].

The microstructures of CS, B-PE, and B-PE/CS complexes were observed using TEM. As shown in Figure 6(c_1_), CS microspheres are tiny fiber shapes, with an average diameter of 0.1–0.2 μm. Furthermore, B-PE microspheres were granular shaped with an average diameter of 0.1–0.2 μm (Figure 6(c_2_)). Remarkably, a tangled mesh structure with a length of about 15–20 μm was observed in the B-PE/CS complexes (Figure 6(c_3_)), further suggesting that B-PE/CS complexes were formed by the bridging effect [57].

**Figure 7:**
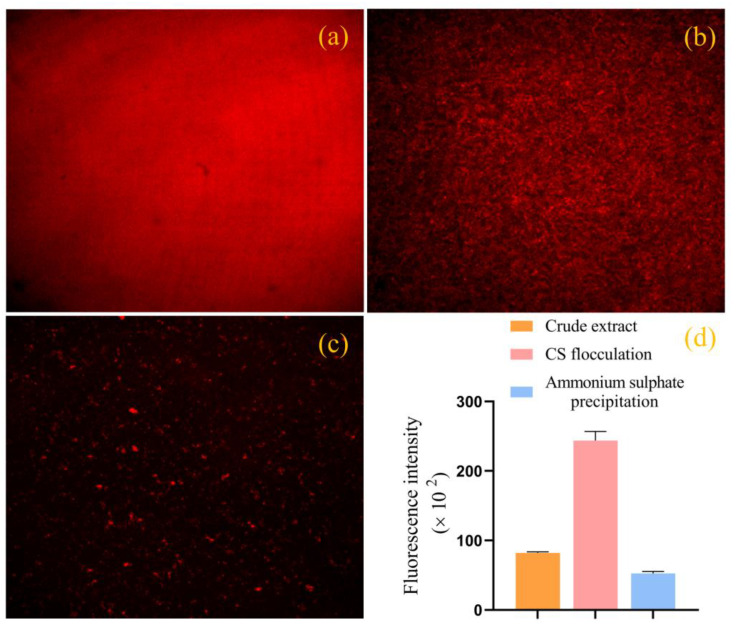
CLSM: (**a**) B-PE; (**b**) B-PE/CS complexes; (**c**) precipitates formed by ammonium sulfate precipitation; (**d**) fluorescence intensity of crude extract, B-PE/CS complexes, and precipitates formed by ammonium sulfate precipitation method.

The degree and density of B-PE and B-PE/CS complexes and precipitates formed by the ammonium sulfate precipitation method were directly observed with a confocal laser scanning microscope (CLSM). As expected, the CLSM image of B-PE/CS complexes revealed that dense and large particle clusters were involved in the flocculation process (Figure 7b). In the case of precipitates formed using the ammonium sulfate precipitation method, the precipitated particle clusters are much smaller and sparser than B-PE/CS complexes are (Figure 7c). Moreover, as shown in Figure 7d, the fluorescence intensity of B-PE/CS complexes was nearly fivefold higher than that of the precipitates formed by the ammonium sulfate precipitation method (2.44 × 10^5^ and 0.53 × 10^5^, respectively). These results suggested that our CS-based flocculation method not only efficiently recovered high-purity B-PE from a low concentration of phycobilin in wastewater, but it also improved the light stability of B-PE, which would promote the application of B-PE as a natural pigment protein in food and chemical applications.

**Figure 8:**
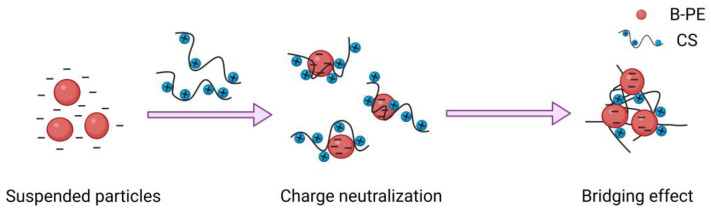
Flocculation mechanism of B-PE and CS.

Based on the above results, we propose the possible flocculation mechanism of B-PE and CS. As shown in Figure 8, first, positively charged CS adsorbed negatively charged B-PE through electrostatic interaction. The surface charges of B-PE were neutralized, resulting in the formation of small flocs. Subsequently, the distance between particles became shorter due to the compression of double electric layer. At this time, CS bound to the B-PE particles had looped and dangling chains that attached to further particles nearby, causing bridging flocculation. Finally, small flocs grouped together to produce large flocs, and a favorable flocculating effect was obtained [58].

## 3. Materials and Methods

### 3.1. Materials

CS samples with different MWs (30, 70, 500, 700, 1200, and 1500 kDa, DD ≥ 95%) were obtained from Cool Chemistry (Beijing, China). Other chemicals, namely, acetic acid, sodium phosphate dibasic dihydrate (Na_2_HPO_4_⋅2H_2_O), sodium phosphate monobasic monohydrate (NaH_2_PO_4_⋅H_2_O), ammonium sulfate ((NH_4_)_2_SO_4_), and sodium chloride (NaCl), were purchased from Macklin Biochemical Co., Ltd. (Shanghai, China). All the reagents used in our experiment were analytically pure. Additionally, deionized water was used.

### 3.2. Preparation of the B-PE Crude Extract

The wastewater used in this experiment was collected from the Marine Biomedical Research Institution (Zhanjiang, China), and it was the remaining liquid after the enrichment of phycobiliproteins. The collected wastewater samples were prefiltered using a 0.45 μm membrane to remove the suspended substances, followed by ultrafiltration by 50 kDa PES membranes in the membrane ultrafiltration system (Guo-chu Tech., China) for desalination. The permeating liquid was referred to as a crude extract. The concentration of B-PE was determined to be 0.04 mg/mL, which was measured using a spectrophotometer (UV 2450, Shimadzu, Japan) and calculated using Equations (1)–(3).

### 3.3. Recovery of B-PE

The experimental procedure for the recovery of B-PE involved two major steps: (i) flocculation by CS; (ii) dissociation by the phosphate buffer.

#### 3.3.1. Flocculation Experiments

The CS solution (0.2% *w*/*v*) was obtained by dissolving the CS powder (30, 70, 500, 700, 1200, and 1500 kDa) in acetic acid (0.25% *v*/*v*), while the remaining solution was refrigerated at 4 °C overnight to ensure complete hydration. The solution was prepared when it was used to avoid hydrolysis.

The desired volume of the CS solution was added to 25 mL of crude extract to obtain the predetermined B-PE/CS mass ratios of 0.025, 0.05, 0.1, 0.2, and 0.4. Subsequently, the pH value of the solution was adjusted to a range of 4.8–6.0 by adding 0.2 M Na_2_HPO_4_ or NaH_2_PO_4_. The solution was gently stirred with a glass rod to achieve the appropriate stirring intensity. Finally, the solution was left to stand for 30 min to allow the complexes to form before centrifuging at 2000 rpm and 4 °C for 15 min. The B-PE concentration (mg/mL) of the supernatant of all samples was determined using Equations (1)–(3). Furthermore, the flocculation efficiency (%) was calculated using Equation (4). Complexes at the bottom were collected and stored at −20 °C for further dissociation experiments.

#### 3.3.2. Dissociation Experiments

Complexes were first added to phosphate buffer (concentrations ranging from 0.1 to 0.5 M; pH ranging from 6.0 to 8.0) and vigorously stirred for 3 min with a vortex mixer (ThermoFisher Scientific, Waltham, MA, USA) to crush the complexes. Subsequently, the tubes were shaken at 500 rpm on a horizontal shaker at 25 °C for 3 h, before centrifuging at 2500 rpm and 4 °C for 15 min. The above procedure was repeated thrice. Finally, the supernatant was collected each time and combined together to determine the B-PE concentration (mg/mL) using Equations (1)–(3). Furthermore, the recovery rate (%) and purity index of B-PE were calculated using Equations (5) and (6), respectively. The CS after dissociation was also collected and combined together, followed by washing with ultrapure water, centrifugation, and drying for reuse.

#### 3.3.3. Ammonium Sulfate Precipitation

The ammonium sulfate precipitation method was used as a control test. Solid ammonium sulfate was added to crude extract to achieve saturation levels of 50% and 80%. The samples were centrifuged at 2000 rpm for 15 min and 8000 rpm/min for 30 min after overnight storage (approximately 12 h) in the refrigerator at 4 °C. The precipitates were collected and suspended in phosphate-buffered saline. The B-PE concentration (mg/mL) of the resulting solution was determined using Equations (1)–(3) after dialyzing with a dialysis bag (MwCO 3500) overnight at 4 °C. The recovery rate (%) and purity index of B-PE were calculated using Equations (5) and (6), respectively.

#### 3.3.4. Analytical Methods

The B-PE concentration was calculated using Equations (1)–(3) [59]:

(1) $$C_{PC}=\frac{A_{615}-0.474\times A_{652}}{5.34}$$

(2) $$C_{APC}=\frac{A_{652}-0.208\times A_{615}}{5.09}$$

(3) $$C_{PE}=\frac{A_{562}-2.41\times (PC)-0.849\times (APC)}{9.62}$$

where C_PC_, C_APC_, and C_PE_ denote phycocyanin, allophycocyanin, and B-PE concentrations (mg/mL), respectively. A_562_, A_615_, and A_652_ represent the absorbance values of the samples at 562, 615, and 652 nm, respectively.

The flocculation efficiency was estimated using Equation (4):

(4) $$Y_{F}\left(\%\right)=1-\frac{C_{2}\times V_{2}}{C_{1}\times V_{1}}$$

where *Y_F_* (%) represents the flocculation efficiency. *C*_1_ and *V*_1_ refer to the B-PE concentration and the total volume of crude extract, respectively. *C*_2_ and *V*_2_ denote the B-PE concentration and the total volume of supernatant after flocculation, respectively.

The recovery rate was determined using Equation (5):

(5) $$Y_{R}\left(\%\right)=\frac{C_{3}\times V_{3}}{C_{1}\times V_{1}}$$

where *Y_R_* (%) denotes the recovery rate. *C*_3_ and *V*_3_ represent the B-PE concentration and the total volume of solution after dissociation or re-dissolution, respectively. *C*_1_ and *V*_1_ represent the B-PE concentration and the total volume of crude extract, respectively.

The purity index was calculated using Equation (6).

(6) $$Purity\;index=\frac{A_{545}}{A_{280}}$$

where *A*_545_ and *A*_280_ represent the absorbance of the samples at 545 and 280 nm, respectively. The former one indicates the amount of B-PE, while the latter one indicates the total amount of proteins [5].

### 3.4. Economic Evaluation

The economic evaluation performed in this study primarily focused on the material consumption and revenues generated by the CS-based flocculation method and ammonium sulfate precipitation method. Recovery costs were calculated per milligram of the recovered B-PE (cost of goods per milligram—CoG/mg). To calculate the recovery costs, the following equation (Equation (7)) was used [46]:

(7) $$\frac{CoG}{mg}=\frac{\sum_{i=1}^{n}\frac{Use\;of\;{material}_{i}}{Batch}\times \frac{Price\;of\;{material}_{i}}{Unit\;of\;{material}_{i}}}{\frac{Amount\;of\;B-PE}{Unit\;of\;crude\;extract}\times Mass\;used\;of\;crude\;extract}$$

For this analysis, the price of the CS was USD 278.57/kg (Cool Chemistry, Beijing, China), and for ammonium sulfate, the price was USD 10.29/kg (Macklin Biochemical Co., Ltd., Shanghai, China). Moreover, the potential revenue mainly depended on the purity index of the recovered B-PE. The purity indices of 3.9 and 0.7 were classified as “drug grade”, with a value of USD 5/mg, and “food-grade”, with a value of approximately USD 0.13/mg, respectively [60,61].

### 3.5. Properties of the Recovered B-PE

The absorption spectra of the recovered B-PE were recorded using a UV/Vis spectrophotometer (UV 2450, Shimadzu, Kyoto, Japan) in the range of 300–700 nm. Fluorescence emission spectra were measured using a fluorescence spectrometer (F-2700, Tokyo, Japan). The fluorescence excitation wavelength was 495 nm, and the scanning range was 530–590 nm, with a slit width of 2.5 nm and an interval of 1 nm. Circular dichroism spectroscopy (Jasco J-800, Easton, MD, USA) was used to determine the secondary structure of the recovered B-PE with phosphate buffer as a baseline correction. The wavelength range was 200–260 nm, with a 1 nm step size and 1 nm bandwidth. The molecular weight of the recovered B-PE was determined using sodium dodecyl sulfate–polyacrylamide gel electrophoresis analysis (SDS-PAGE). The concentrated adhesive and separated adhesive had values of 10% and 5%, respectively. Electrophoresis was carried out at 50 V for 3–4 h. The proteins were stained with Coomassie Brilliant Blue R250 and destained with deionized water.

### 3.6. Study of Flocculation Mechanism

The complexes were prepared at different B-PE/CS mass ratios (0.025, 0.05, 0.1, 0.2, and 0.4) and solution pH (4.8, 5.1, 5.4, 5.7, and 6.0). Additionally, their ζ potentials of were monitored at room temperature by Malvern Ζsizer (Nano ZS 90, Worcestershire, UK). B-PE, CS, and their complexes were freeze dried to prepare solid samples for Fourier transform infrared spectrometry (FTIR), scanning electron microscopy (SEM), and transmission electron microscopy (TEM). FTIR spectra were recorded on an FTIR spectrometer (IRAffinity-1S, Kyoto, Japan) at the range of wavelength 400–4000 cm^−1^. Two mg of finely ground solid samples was mixed with potassium bromide and compressed into thin, transparent pellets for FTIR testing. The external morphology of samples was observed using a scanning electron microscope (JSM-6700F, JEOL, Kyoto, Japan) after gold spraying on the surface of 10 mg samples. Microstructures of samples (3 mg) were detected using the HT-7700 transmission electron microscope (Hitachi, Kyoto, Japan). A confocal laser scanning microscope (CLSM, IXplore SpinSR, Kyoto, Japan) was used to observe the microstructures of B-PE/CS complexes and precipitates formed by the ammonium sulfate precipitation method. The excitation wavelength was 561 nm, and the magnification was 40×.

### 3.7. Statistical Analyses

All measurements were carried out in triplicate. One-way analysis of variance (ANOVA) and Student’s *t*-test were used to compare the significance of the results obtained (*p* < 0.05). Statistical analyses were performed using SPSS software version 25.0.

## 4. Conclusions

In this study, we developed a CS-based flocculation method for the efficient recovery of B-PE from a low concentration of phycobilin in wastewater. Based on optimal recovery conditions, the maximum flocculation efficiency of CS, recovery rate, and purity index of B-PE were up to 97.19% ± 0.59%, 72.07% ± 1.37%, and 3.20 ± 0.025 (drug grade), respectively. Compared with the ammonium sulfate precipitation method (control method), our CS-based flocculation method offered many substantial improvements in the recovery rate and purity index of B-PE, in addition to cost-effectiveness. Thus, this work provided an efficient and economical method to recover B-PE with a high purity from a low concentration of phycobilin in wastewater, which facilitates the use of B-PE in the food, pharmaceutical, and cosmetic industries.

## Figures and Tables

**Table 1:** Comparison between CS-based flocculation method and ammonium sulfate precipitation method *.

Items	CS-Based Flocculation Method	80% Saturated Ammonium Sulfate Solution	50% Saturated Ammonium Sulfate Solution
Centrifugal speed (rpm)	2000	8000	8000
Centrifugal time (min)	15	30	30
Dialysis	No	Yes	Yes
Purity index	3.20 ± 0.025	0.62 ± 0.021	0.83 ± 0.018
Recovery rate (%)	72.07 ± 1.37	45.10 ± 2.28	36.13 ± 2.44

* Data are expressed as the mean ± SD (*n* = 3).

**Table 2:** Economic dataset used for economic evaluation *.

Item	CS-Based Flocculation Method	Ammonium Sulfate Precipitation Method
Material price (USD per kg)	278.57	10.29
Material consumption (kg)	1.39 × 10^−5^	3.10 × 10^−2^
Purity index	3.20 ± 0.025	0.62 ± 0.021
Total costs (USD)	3.90 × 10^−3^	3.10 × 10^−1^
Revenues generated (USD)	4.10	0.12

* Data are expressed as the mean ± SD (*n* = 3).

## Data Availability

Data are contained within the article.

## Sample Availability

Samples of the compounds are available from the authors upon reasonable request.
